# The role of mGluR5 on the therapeutic effects of ketamine in Wistar rats

**DOI:** 10.1007/s00213-024-06571-3

**Published:** 2024-03-09

**Authors:** Dilan Gokalp, Gunes Unal

**Affiliations:** Behavioral Neuroscience Laboratory, Department of Psychology, Boğaziçi University, 34342 Istanbul, Turkey

**Keywords:** Ketamine, mGluR5, Behavioral despair, Anxiety, Fear, FST, OFT, EPM, MTEP, CDPPB

## Abstract

**Rationale:**

Ketamine produces dissociative, psychomimetic, anxiolytic, antidepressant, and anesthetic effects in a dose dependent manner. It has a complex mechanism of action that involve alterations in other glutamate receptors. The metabotropic glutamate receptor 5 (mGluR5) has been investigated in relation to the psychotic and anesthetic properties of ketamine, while its role in mediating the therapeutic effects of ketamine remains unknown.

**Objectives:**

We investigated the role of mGluR5 on the antidepressant, anxiolytic and fear memory-related effects of ketamine in adult male Wistar rats.

**Methods:**

Two sets of experiments were conducted. We first utilized the positive allosteric modulator CDPPB to investigate how acute mGluR5 activation regulates the therapeutic effects of ketamine (10 mg/kg). We then tested the synergistic antidepressant effect of mGluR5 antagonism and ketamine by combining MTEP with a sub-effective dose of ketamine (1 mg/kg). Behavioral despair, locomotor activity, anxiety-like behavior, and fear memory were respectively assessed in the forced swim test (FST), open field test (OFT), elevated plus maze (EPM), and auditory fear conditioning.

**Results:**

Enhancing mGluR5 activity via CDPPB occluded the antidepressant effect of ketamine without changing locomotor activity. Furthermore, concomitant administration of MTEP and ketamine exhibited a robust synergistic antidepressant effect. The MTEP + ketamine treatment, however, blocked the anxiolytic effect observed by sole administration of MTEP or the low dose ketamine.

**Conclusions:**

These findings suggest that suppressed mGluR5 activity is required for the antidepressant effects of ketamine. Consequently, the antagonism of mGluR5 enhances the antidepressant effectiveness of low dose ketamine, but eliminates its anxiolytic effects.

## Introduction

Ketamine is a non-competitive N-methyl D-aspartate receptor (NMDAR) antagonist with rapid and sustained antidepressant effects (Zarate et al. 2006). It has also demonstrated potential in alleviating symptoms of anxiety (Silote et al. 2020; Truppman Lattie et al. 2021) and in the modulation of fear memory (Choi et al. 2020; Silote et al. 2020). The antidepressant effects of ketamine, initially observed in rodents in (Sofia and Harakal 1975), have been investigated in both animal models (Akan et al. 2023; Ecevitoglu et al. 2019; Kingir et al. 2023; Yilmaz et al. 2002) and clinical settings (Arabzadeh et al. 2018; Lapidus et al. 2014; Zarate et al. 2006) by utilizing diverse doses and administration methods. However, the therapeutic benefits are often accompanied by psychomimetic and dissociative side effects (Cooper et al. 2017). These diverse side effects stem from ketamine’s complex mechanism of action, extending beyond the antagonism of the NMDAR (Kim et al. 2023; Zanos and Gould 2018). The administration of ketamine triggers a cascade of downstream effects, including the attenuation (Piva et al. 2020) and metabolic activity changes of metabotropic glutamate receptor 5 (mGluR5) (DeLorenzo et al. 2015; Esterlis et al. 2018). However, the specific role of mGluR5 in mediating the rapid therapeutic effects of ketamine remains unknown. This study aims to elucidate the contribution of mGluR5 to the antidepressant, anxiolytic and fear memory-related effects of ketamine in adult male Wistar rats by combining a positive allosteric modulator (PAM) and an antagonist of mGluR5 with antidepressant and low doses of ketamine, respectively.

The fast-onset antidepressant action of ketamine is attributed to the blocking the NMDARs of GABAergic interneurons, which in turn leads to disinhibition of principal cells in various limbic structures (Widman and McMahon 2018; Zanos and Gould 2018; Zhang et al. 2021). Resulting increase in glutamate release is associated with the activation (Fukumoto et al. 2016; Koike et al. 2011) and phosphorylation (Maeng et al. 2008; K. Zhang et al. 2017) of the α-amino-3-hydroxy-5-methyl-4-isoxazolepropionic acid (AMPA) receptor (Zhang et al. 2016). The mechanism of action of ketamine, therefore, starts with non-competitive antagonism of the NMDARs and subsequently leads to the modulation of other glutamatergic receptors. In addition to the well-studied AMPA receptors, the metabotropic glutamate receptors are also investigated for their role in the therapeutic effects of ketamine. Group II mGluR antagonism led to a synergistic antidepressant effect when combined with a sub-effective dose of hydroxynorketamine (Zanos et al. 2019) or ketamine (Pałucha-Poniewiera et al. 2019; Podkowa et al. 2016). The role of predominantly postsynaptic mGluR5s, however, has been mostly studied in relation to the anesthetic and psychotic properties of ketamine (Krystal et al. 2010). Pre-treatment with mGluR5 agonist CHPG reduced the duration of ketamine-induced anesthesia, while its antagonist MPEP increased this duration (Sou et al. 2006). Likewise, mGluR5 agonism reversed ketamine-induced schizophrenia-like behaviors in mice (Chan et al. 2008). The sole study investigating mGluR5 and ketamine in relation to depression showed that ketamine reversed depressive-like behavior induced by viral overexpression of mGluR5 in the hippocampus (Wang et al. 2020).

The mGluR5 is highly expressed in the hippocampus, cerebral cortex, olfactory bulb, caudate/putamen, and lateral septum in the rat brain (Romano et al. 1995; Su et al. 2022). They are usually localized in the postsynaptic membrane (Niswender and Conn 2010) and interact with the NMDARs via several molecular mechanisms (Chen et al. 2011; Jin et al. 2013; Matta et al. 2011). The activation of mGluR5 potentiates NMDAR currents (Awad et al. 2000) via protein kinase C (Benquet et al. 2002), while activation of the NMDAR leads to phosphorylation and potentiation of mGluR5s (Alagarsamy et al. 1999, 2002, 2005). This functional coupling between the NMDAR and mGluR5 arises from the physical association between these receptors on the postsynaptic membrane. The Shank proteins of the PSD-95 complex of the NMDAR are coupled to the Homer proteins of the mGluR5 (Tu et al. 1999). Homer1b/c, the long form of Homer1, forms connections with the NMDAR Shank protein, and contributes to the facilitation of NMDAR activity (Tu et al. 1999). In contrast, Homer1a, the short form of Homer1, leads to the uncoupling of the long Homer proteins (Bockaert et al. 2021) and the subsequent disruption of the mGluR5-NMDAR interaction (Clifton et al. 2019). Notably, ketamine decreases the transcription of Homer1b/c (de Bartolomeis et al. 2013), and increases Homer1a (de Bartolomeis et al. 2013; Serchov et al. 2015), suggesting that suppressed mGluR5 activity may contribute its cognitive and affective properties.

We investigated the role of mGluR5 in the therapeutic effects of ketamine in two sets of experiments. We first utilized the positive allosteric modulator CDPPB (10 mg/kg) to investigate how acute mGluR5 activation regulates the rapid therapeutic effects of an antidepressant dose of ketamine (10 mg/kg). We have then tested whether antagonism of the mGluR5 could be used to enhance the ameliorative effects of ketamine via a synergistic mechanism (Chaki and Watanabe 2023). For this purpose, we have combined low doses of the mGluR5 antagonist MTEP (1.25 mg/kg; Belozertseva et al. 2007) and ketamine (1 mg/kg; Podkowa et al. 2016), and tested the effects of this cocktail drug on behavioral despair, anxiety-like behavior, and fear extinction.

## Materials and methods

### Subjects

Sixty-six experimentally naïve adult male Wistar rats (3–6 months old) were used for the experiments. The animals were transferred to individual cages and housed under standard laboratory conditions (21°C, ~ 50–60% humidity; 12:12 day/night cycle with lights on at 08:00), and provided with ad libitum food and water throughout the experiment. Fifty-six animals were divided into experimental groups (*n* = 8 per group) according to their initial weights and used in the main study. The remaining 10 animals were used in a control experiment (refer to Pharmacological Agents). All procedures were approved by the Boğaziçi University Ethics Committee for the Use of Animals in Experiments (permission no: 2021–015).

### Experimental groups

Two groups of experiments were conducted to investigate the role of mGluR5 on the therapeutic action of ketamine, and reveal potential synergistic effects of mGluR5 antagonism with ketamine. First, an antidepressant dose of ketamine (10 mg/kg) was combined with the mGluR5 PAM CDPPB (3-cyano-N-(1,3-diphenyl-1H-pyrazol-5-yl) benzamide, 10 mg/kg) in order to elucidate the role of mGluR5s in ketamine’s mechanism of action, and investigate if the antidepressant effect of ketamine would be abolished due to allosteric modulation of the mGluR5. The animals were divided into three groups: a ketamine only group (saline + ketamine; Ket10, *n* = 8); a CDPPB only group (CDPPB + saline; CDPPB, *n* = 8); and a combinatorial treatment group (CDPPB + Ket10, *n* = 8). In the second set of experiments, we examined the synergistic therapeutic effects of the mGluR5 antagonist MTEP with a low (i.e. not antidepressant) dose of ketamine (1 mg/kg). This experiment was conducted using a low dose of ketamine, as administering the antidepressant dose (10 mg/kg) could result in a ceiling effect, potentially obstructing the observation of the combinatorial or synergistic effects under investigation. The animals were separated into saline + ketamine (Ket1, *n* = 8), MTEP + saline (MTEP, *n* = 8), and MTEP + ketamine (MTEP + Ket1, *n* = 8) groups. In addition, a saline + saline (vehicle) group was added as a true control for both experiments (*n* = 8).

### Experimental design

**Fig. 1:**
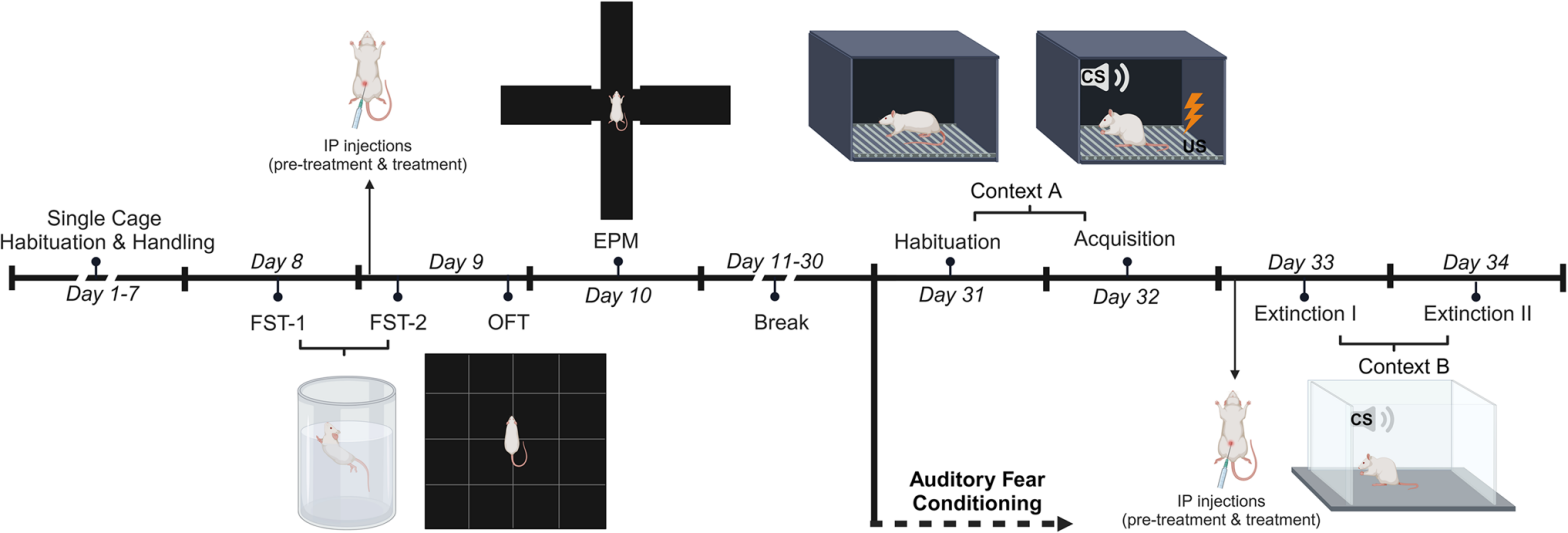
Experimental timeline showing the schedule of behavioral experiments and drug administrations. IP, intraperitoneal; FST, forced swim test; OFT, open field test; EPM, elevated plus maze; CS, conditioned stimulus; US, unconditioned stimulus

All groups followed identical experimental procedures in the same sequence. Animals were acclimated to individual cages for a week, with daily handling to reduce stress during subsequent drug administration. Behavioral testing commenced after a week of single-cage acclimation and handling (Fig. 1). The forced swim test (FST) was conducted on Day 8 and Day 9. On Day 9, animals received two intraperitoneal (IP) drug injections 40 and 30 min before the test session of FST (FST-2) (refer to Pharmacological Agents). The FST was followed by the open field test (OFT) conducted 6 h after drug administrations. The elevated plus maze (EPM) was conducted on Day 10. A 3-week-long break was given after the EPM in order to mitigate the behavioral effects of pharmacological treatments before the habituation session of auditory fear conditioning on Day 31. During the last five days of this break, animals were handled again to prepare them for the upcoming IP injections. Fear conditioning took place on Day 32, which was followed by two extinction sessions in a novel context on Day 33 and Day 34. Pharmacological treatments were repeated 40 and 30 min before fear extinction on Day 33 (Fig. 1). All behavioral procedures were carried out between 9:00 and 19:00 in the same behavioral testing room.

### Pharmacological agents

Ketamine hydrochloride (Keta-control, Doğa İlaç, 100 mg/ml, Istanbul, Turkey) was diluted in 0.9% saline to prepare both the effective antidepressant dose (10 mg/kg, IP) and the sub-effective dose (1 mg/kg, IP). CDPPB (10 mg/kg, IP; MedChemExpress, Sollentuna, Sweden), was dissolved in a 10% DMSO and 90% corn oil solution, while MTEP (1.25 mg/kg, IP; MedChemExpress, Sollentuna, Sweden) was dissolved in 0.9% saline containing 2% Tween 80. Sterile saline served as the vehicle for all drugs. In order to ensure that 10% DMSO in the CDPPB groups does not cause inflammation and affect behavior, we conducted a control experiment comparing the effects of 10% DMSO/90% corn oil (*n* = 5) and saline (*n* = 5) in the FST, OFT and EPM. The timing of the injections and behavioral tests in this experiment followed that of the main study.

CDPPB and MTEP were administered 40 min before the FST-2 and the first day of fear extinction, while ketamine doses were administered 30 min before behavioral testing (i.e. 10 min after CDPPB or MTEP). The injection times were selected to ensure that the peak effect of each drug coincided with the first behavioral test. All drugs, except for MTEP, were administered at a volume of 1 ml/kg, while MTEP was administered at a volume of 2 ml/kg.

### Forced Swim Test (FST)

The Forced Swim Test (FST) is used to assess behavioral despair and antidepressant effects by primarily measuring immobility duration during the test session (Unal and Canbeyli 2019). We followed the standard two-day protocol by Porsolt et al. (1977). Animals were placed into a cylindrical acrylic chamber (height = 45 cm, r = 15 cm) filled with water (23 ± 1 °C) to a depth of 30 cm. At the beginning of each behavioral test, the animals were acclimatized to the test room for 5 min. The training session of FST (FST-1) lasted for 15 min, which was followed by the 5-min test session (FST-2) 24 h later. After the test, animals were dried under an infrared heat lamp and returned to their cages. The durations of immobility, swimming, and struggling were analyzed using EthoVision XT 17 (Noldus, Wageningen, NL). Researchers, blind to the experimental conditions, recorded the number of diving and headshakes. These episodic responses may not correlate with other FST behaviors or antidepressant treatment (Cryan et al. 2005), but they reflect differences in hormonal levels (Kokras et al. 2017).

### Open Field Test (OFT)

General locomotor activity was measured in an opaque black square apparatus (70 × 70 × 45 cm) (Valle 1970). The arena was divided virtually into a center compartment (45 × 45 cm; 100 ± 5 lx) and a peripheral area (60 ± 5 lx) to evaluate anxiety-like behavior. Following a 5-min acclimation to the testing environment, each animal was placed in the center of the apparatus for a 5-min observation period. We recorded and analyzed the total duration of locomotor activity, as well as the time spent in both the center and peripheral areas of the maze using EthoVision XT 17 (Noldus, Wageningen, NL). The time spent at the brighter center compartment of the maze is associated with anxiolytic effects (Fraser et al. 2010). Frequency of supported and unsupported rearing were also recorded. Supported rearing involves touching the maze walls with forelimbs, while unsupported rearing was defined as standing on the hindlimbs without touching the maze walls. The maze was cleaned with 70% ethanol between sessions to remove any olfactory cues for the next animal.

### Elevated Plus Maze (EPM)

We used a wooden plus-shaped apparatus with two acrylic transparent (open) and two opaque (closed) arms (arm length = 50 cm, width = 10 cm), positioned 50 cm above the floor. The open arms had a light intensity of 260 ± 10 lx, while the closed arms had 60 ± 10 lx. Animals were introduced into the center of the Elevated Plus Maze (EPM), facing either an open or closed arm, with the orientation balanced within and across groups. Each session lasted 5 min, following the protocol by Walf and Frye (2007). We measured and analyzed the time spent in the open and closed arms, as well as the total duration of locomotor activity using EthoVision XT 17 (Noldus, Wageningen, NL). The time spent in the closed arms is correlated with anxiety, while open arm preference indicates anxiolytic effects (Walf and Frye 2007).

### Auditory fear conditioning

We employed a modified version of the auditory fear conditioning protocol adapted from Burghardt et al. (2004). The habituation and acquisition sessions were carried out in a fear conditioning chamber (Context A; 21 × 45 × 27 cm) under bright lighting. The chamber featured a floor composed of 32 parallel metal bars spaced 1.4 cm apart, with transparent dark grey acrylic walls and a matching acrylic lid. On Day 29, animals were habituated to Context A for 20 min to reduce novelty stress. On Day 30, a 3-min acclimation period was followed by two pairings of an auditory conditioned stimulus (CS; 75 dB, 2 kHz, 20 s) with a mild footshock (unconditioned stimulus, US; 0.7 mA, 2 s, onset: 0 s after CS offset, 90–120 s ITI). The apparatus was cleaned with 70% ethanol between sessions to remove olfactory cues for the next animal.

Fear extinction was conducted in a novel chamber (Context B; 41 × 43 × 31 cm), an open-top apparatus with a transparent acrylic wall and opaque black floor, under dim light. It was cleaned with 1% peppermint oil between sessions to differentiate odors from Context A. Fear Extinction I began after drug administration, with a 3-min acclimation period to Context B, followed by 10 CS presentations (75 dB, 2 kHz, 20 s, 90–120 s ITI). After 24 h, the same procedure was repeated for Fear Extinction II. Freezing behavior, defined as immobility except for respiration, was analyzed using the freezing module of ezTrack (Pennington et al. 2019). The 3-min acclimation period was used to measure baseline freezing. Conditional response was determined by calculating the freezing percentage during auditory cue presentations. Furthermore, we analyzed general locomotor activity during Fear Extinction I to demonstrate acute changes in locomotion as a result of pharmacological interventions. Rearing and grooming behaviors were also recorded during the extinction sessions.

### Statistical analyses

Data analyses were performed using GraphPad Prism 9.0. The effects of the antidepressant and low dose ketamine were evaluated in comparison to the vehicle (saline) group through the application of Student’s t-tests. In each experiment, two-way or three-way ANOVAs were used to reveal main treatment effects and interactions. Subsequent post-hoc tests compared the experimental and vehicle groups. We applied Sidak’s correction to account for a single significant main effect, and for significant main effects and their interactions, we utilized Sidak’s multiple comparisons. Statistical significance was considered at* p* < 0.05. Data is presented as mean ± SEM in the figures.

## Results

### DMSO control experiment

The DMSO group (*M* = 17.75, *SD* = 13.63) and saline group (*M* = 23.86, *SD* = 17.24) showed similar immobility times in FST-2 (*t*(8) = 0.62, *p* = 0.55, Student’s t-test). There were also no differences in locomotor activity (*t*(8) = 0.55, *p* = 0.59) or time spent in the center of the OFT (*t*(8) = 0.63, *p* = 0.54, Student’s t-test). In addition, both groups displayed similar levels of anxiety in the EPM (*t*(8) = 0.77, *p* = 0.46, Student’s t-test). These findings are in line with earlier behavioral studies that utilize saline and DMSO or Tween-80 as vehicle groups, and demonstrate comparable durations in locomotor activity (Amiri et al. 2015; Castro et al. 1995; Jesse et al. 2008; Konieczny et al. 1998) and behavioral despair (Amiri et al. 2015; Tanyeri et al. 2013; Zomkowski et al. 2005).

### Behavioral despair

We confirmed the antidepressant effect of the 10 mg/kg dose of ketamine (i.e. Ket10 group) in the FST, which produced a significant reduction in immobility (*M* = 18.23, *SD* = 11.18) compared to the vehicle group (*M* = 44.49, *SD* = 21.90; *t*(14) = 3.01, *p* = 0.009, *d* = 1.51, Student’s t-test). In contrast, the sub-effective dose used in the Ket1 group led to similar immobility levels (*M* = 31.54, *SD* = 26.42) with the control animals (*t*(14) = 1.06, *p* = 0.304, Student’s t-test).

We analyzed FST-1 by dividing it into three 5-min periods to confirm the induction of behavioral despair. We found a significant increase in immobility behavior over time (*F*(2,110) = 98.07, *p* < 0.001, *R*^*2*^ = 0.64, repeated measures one-way ANOVA), consistent with earlier protocols (Porsolt et al. 1977; Yankelevitch-Yahav et al. 2015). Immobility time during the last five minutes (*M* = 78.53, *SD* = 38.39) was higher than in the second five minutes (*M* = 62.59, *SD* = 29.43; *t*(110) = 4.00, *p* < 0.001, *d* = 0.46), and the first five minutes (*M* = 24.27, *SD* = 14.69; *t*(110) = 13.62, *p* < 0.001, *d* = 1.86). Similarly, immobility time significantly increased from the first five minutes to the second five minutes of FST-1 (*t*(110) = 9.62, *p* < 0.001, *d* = 1.64, Sidak’s corrected). We also compared the immobility time in the first five minutes of FST-1 to FST-2 for each group, and found that MTEP led to an elevated immobility duration in FST-2 (*M* = 37.35, *SD* = 21.00) compared to the initial phase of FST-1 (*M* = 20.91, *SD* = 8.91; *t*(7) = 3.04, *p* = 0.019, Student’s t-test).

**Fig. 2:**
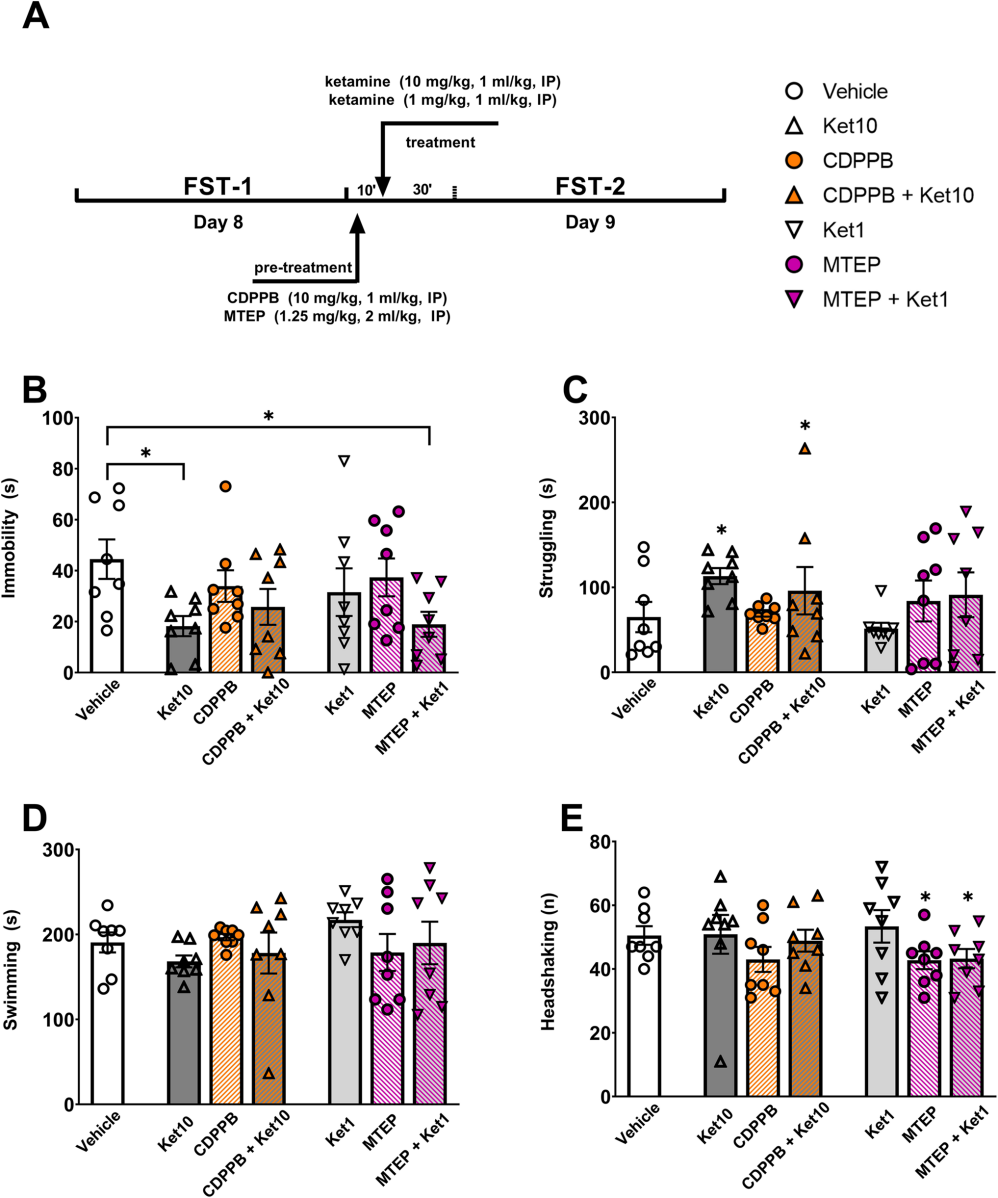
The forced swim test (FST). **A**) The experimental design and treatment schedule of the FST, and the legend for all experimental groups. **B**) The duration of immobility. **C**) The duration of struggling behavior. **D**) The duration of swimming. **E**) The number of headshaking behaviors. Asterisks denote statistically significant (*p* < .05) main effects and post-hoc comparisons (brackets). Error bars represent SEM

In following experiments, pre-treatment with mGluR5 agents was done 10 min before ketamine administration as explained earlier (Fig. 2A). We observed a significant main effect of ketamine following mGluR5 activation via CDPPB pre-treatment (*F*(1,28) = 12.90, *p* = 0.001, *R*^*2*^ = 19.41, 2 × 2 two-way ANOVA; Fig. 2B), as the Ket10 group displayed less immobility compared to the vehicle group (*M* = 44.49, *SD* = 21.90; *t*(28) = 2.90, *p* = 0.021). The immobility scores of CDPPB + Ket10 (*M* = 25.76, *SD* = 19.93) did not differ from the vehicle group (*M* = 44.49, *SD* = 21.90), *t*(28) = 2.07, *p* = 0.137) or the Ket10 group (*t*(28) = 0.83, *p* = 0.797, Sidak’s corrected; Fig. 2B), suggesting a partial attenuation (Koike et al. 2011; Li et al. 2015) or occlusion (Gerhard et al. 2020) of the antidepressant effect of ketamine by CDPPB pre-treatment.

The combination of the sub-effective dose of ketamine (1 mg/kg) with MTEP (1.25 mg/kg) also revealed the effect of ketamine (*F*(1,28) = 4.35, *p* = 0.046, *R*^*2*^ = 12.72; Fig. 2B). The MTEP + Ket1 group (*M* = 18.93, *SD* = 13.85) exhibited significantly less immobility compared to the vehicle group (*M* = 44.49, *SD* = 21.90; *t*(28) = 2.40, *p* = 0.046,* d* = 1.39; Fig. 2B). In contrast, animals receiving MTEP alone (M = 37.35, SD = 20.99) showed a similar immobility duration to the vehicle group (*M* = 44.49, *SD* = 21.90). Likewise, the Ket1 group (*M* = 31.54, *SD* = 26.42) did not differ from the vehicle group (*M* = 44.49, *SD* = 21.90;* t*(28) = 1.21, *p* = 0.413, Sidak’s corrected). These findings collectively indicate a synergistic antidepressant effect of MTEP and ketamine.

The struggling behavior observed in the FST was affected by the administration of ketamine (10 mg/kg) (*F*(1,28) = 4.67, *p* = 0.039, *R*^*2*^ = 14.08, 2 × 2 two-way ANOVA). However, CDPPB (*F*(1,28) = 0.13, *p* = 0.713) or its interaction with ketamine (*F*(1,28) = 0.38 *p* = 0.539) did not have an impact on the struggling behavior. Similarly, MTEP or ketamine (1 mg/kg) did not produce a main effect (*F*(1,28) = 2.10, *p* = 0.158 and *F*(1,28) = 0.02, *p* = 0.876, respectively) or revealed a significant interaction (*F*(1,28) = 0.26, *p* = 0.61, 2 × 2 two-way ANOVA; Fig. 2C).

Swimming performance in the FST remained unaffected by the presence of mGluR5 agents or ketamine. There was no main effect of CDPPB (*F*(1,28) = 0.33, *p* = 0.569, 2 × 2 two-way ANOVA) or ketamine (10 mg/kg; *F*(1,28) = 2.14, *p* = 0.155) or their interaction (*F*(1,28) = 0.01, *p* = 0.897) on swimming duration. Likewise, we found no main effect of MTEP (1.25 mg/kg; *F*(1,28) = 1.15, *p* = 0.293), ketamine (1 mg/kg; *F*(1,28) = 1.08, *p* = 0.308), or their interaction (*F*(1,28) = 0.18, *p* = 0.674, 2 × 2 two-way ANOVA; Fig. 2D).

The frequency of diving and headshaking behavior were also not influenced by the administration of CDPPB or ketamine (all *p* values > 0.5, 2 × 2 two-way ANOVA). In the MTEP experiments, diving behavior did not show a dependence on MTEP (*F*(1,28) = 0.22, *p* = 0.637), ketamine (1 mg/kg; *F*(1,28) = 1.53, *p* = 0.225), or their interaction (*F*(1,28) = 0.44, *p* = 0.510, 2 × 2 two-way ANOVA). In contrast, pre-treatment with MTEP had a significant effect on headshaking (*F*(1,28) = 6.20, *p* = 0.019, *R*^*2*^ = 17.96, 2 × 2 two-way ANOVA; Fig. 2E).

### Locomotor activity and anxiety-like behavior

**Fig. 3:**
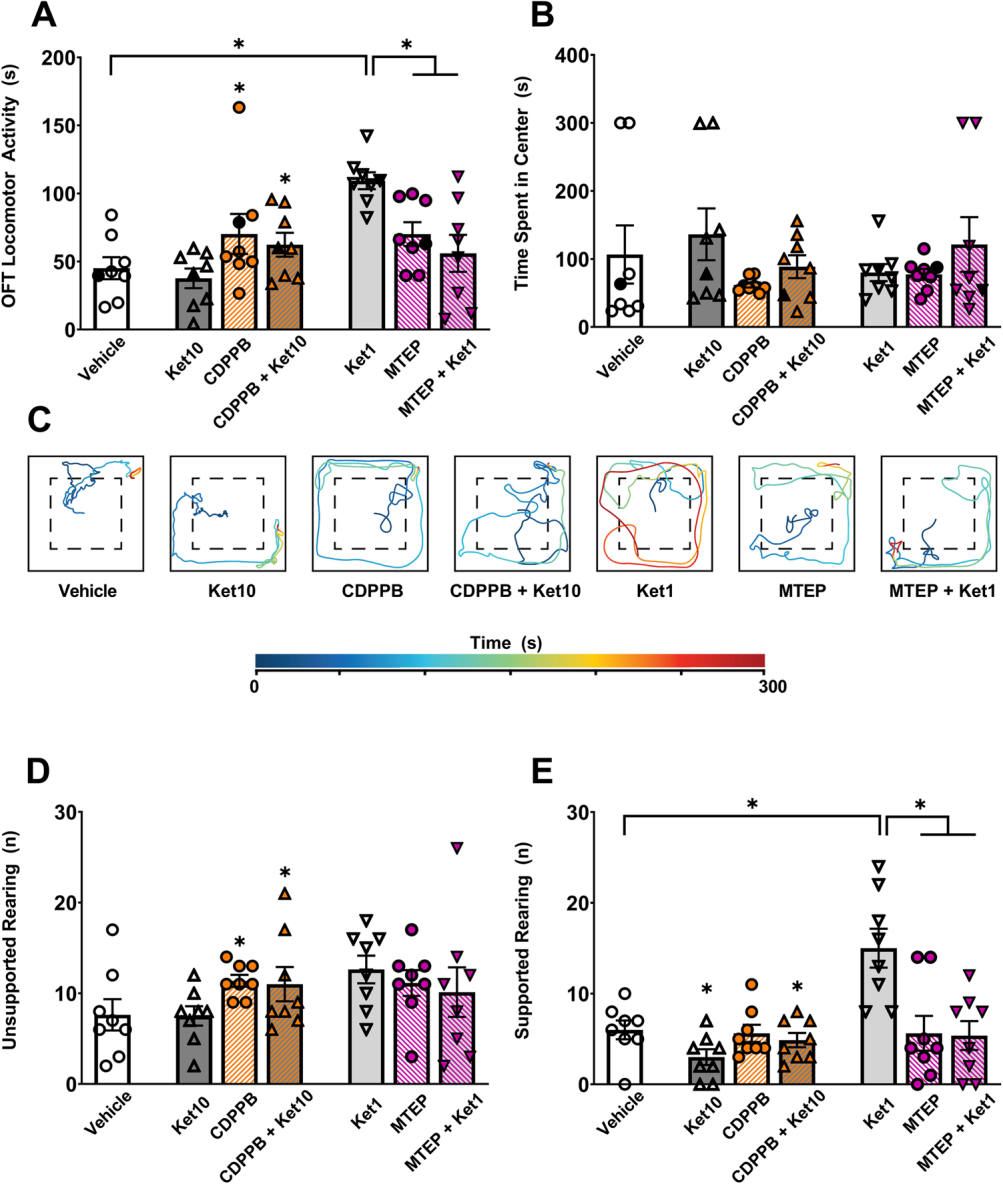
The open filed test (OFT). **A**) The duration of overall locomotor activity. **B**) The time spent in the center zone of the maze. **C**) Movement trajectories of representative animals (black data points in Panels A and B) from each group. **D**) The number of unsupported rearing. **E**) The number of supported rearing. Asterisks denote statistically significant (*p* < .05) main effects and post-hoc comparisons (brackets). Error bars represent SEM

The activation of mGluR5 via CDPPB elevated locomotor activity (*F*(1,28) = 6.02, *p* = 0.021, *R*^*2*^ = 17.41; Fig. 3A). This observation was not affected by ketamine (10 mg/kg; *F*(1,28) = 0.57, *p* = 0.456), and there was no interaction between CDPPB and ketamine (*F*(1,28) = 0, *p* = 0.984). In the MTEP experiments, in contrast, ketamine (1 mg/kg) exerted a main effect on locomotor activity (*F*(1,28) = 6.87, *p* = 0.014, *R*^*2*^ = 12.76), along with an interaction between ketamine and MTEP (*F*(1,28) = 16.81, *p* < 0.001, *R*^*2*^ = 31.21, 2 × 2 two-way ANOVA). The Ket1 group (*M* = 109.40, *SD* = 17.54) displayed hyperlocomotion compared to the vehicle group (*M* = 45.10, *SD* = 22.92; *t*(28) = 4.75, *p* < 0.001, *d* = 3.15). Importantly, combinatorial treatment of MTEP and ketamine (*M* = 56.03, *SD* = 38.46) restored locomotion to baseline (vehicle) levels (*t*(28) = 0.88, *p* = 0.964) and prevented the hyperlocomotion observed in ketamine receiving animals (*t*(28) = 3.94, *p* = 0.003, *d* = 1.78; Sidak’s corrected, Fig. 3A).

The time spent in the center of the OFT was not influenced by mGluR5 agents or ketamine (all *p* values > 0.5, 2 × 2 two-way ANOVA; Fig. 3B). Accordingly, all groups exhibited comparable levels of thigmotaxis (Fig. 3C). Rearing behavior, in contrast, differed among experimental conditions. CDPPB activation of mGluR5 had a main effect on unsupported rearing behavior (*F*(1,28) = 6.487, *p* = 0.017, *R*^*2*^ = 18.79, 2 × 2 two-way ANOVA; Fig. 3D). In the case of supported rearing, the administration of ketamine (10 mg/kg) exhibited a main effect (*F*(1,28) = 4.21, *p* = 0.049, *R*^*2*^ = 12.25, 2 × 2 two-way ANOVA; Fig. 3E). The low dose of ketamine (*F*(1,28) = 6.44, *p* = 0.01, *R*^*2*^ = 12.87) and MTEP also had a main effect (*F*(1,28 = 8.41, *p* = 0.007, *R*^*2*^ = 16.71) on supported rearing, with a significant interaction (*F*(1,28) = 7.19, *p* = 0.012, *R*^*2*^ = 14.38, 2 × 2 two-way ANOVA; Fig. 3E). The Ket1 group (*M* = 15.00, *SD* = 6.07) displayed higher frequency of supported rearing compared to the vehicle group (*M* = 6.00, *SD* = 2.92; *t*(28) = 3.69, *p* = 0.006, *d* = 2.09), MTEP group (*M* = 5.62, *SD* = 5.42; *t*(28) = 3.84, *p* = 0.004, *d* = 1.63), and the MTEP + Ket1 group (*M* = 5.37, *SD* = 4.50; *t*(28) = 3.94, *p* = 0.003,* d* = 1.80; Fig. 3E).

**Fig. 4:**
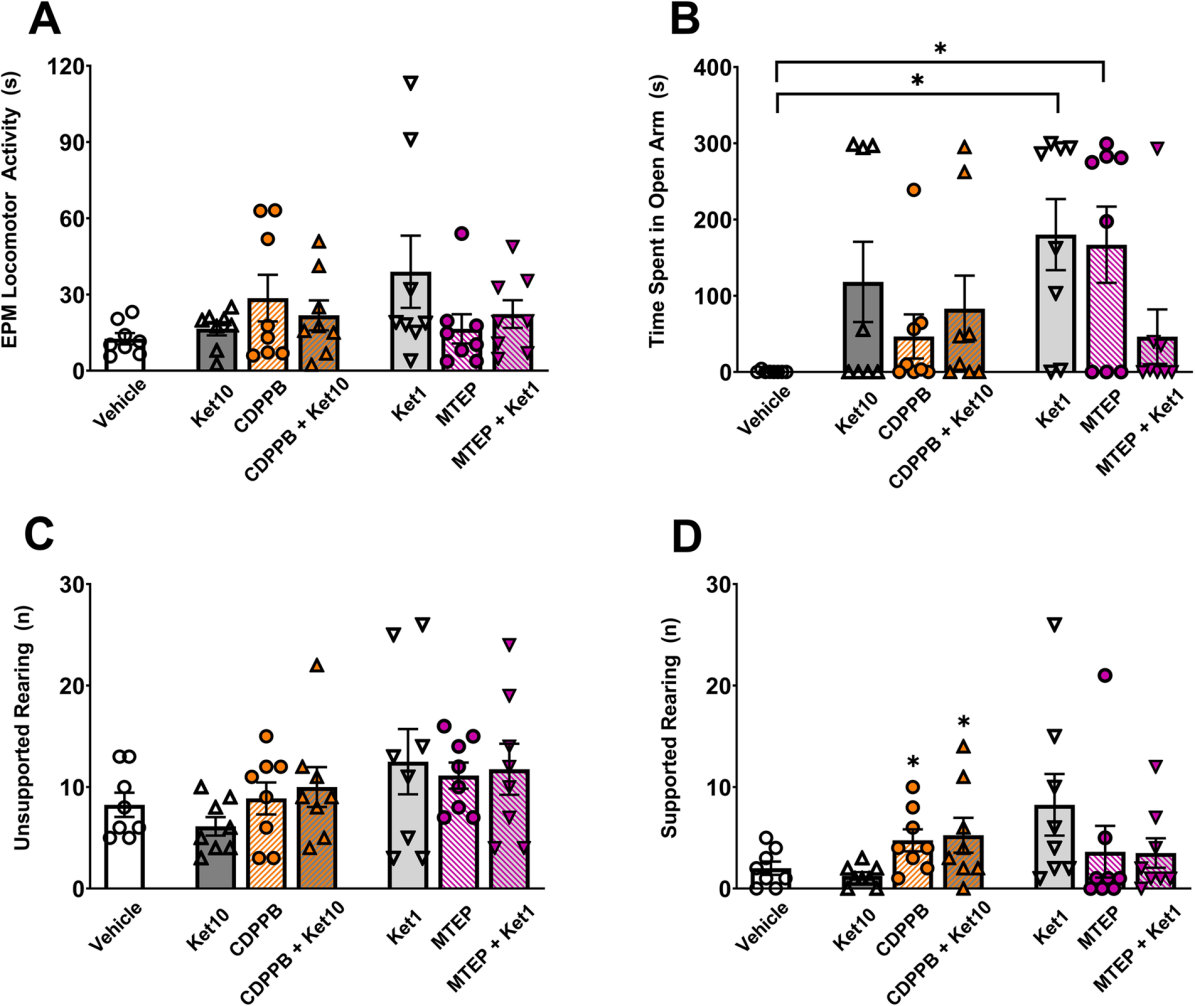
The elevated plus maze (EPM). **A**) The duration of overall locomotor activity. **B**) The time spent in the open arms of the maze. **C**) The number of unsupported rearing. **D**) The number of supported rearing. Asterisks denote statistically significant (*p* < .05) main effects and post-hoc comparisons (brackets). Error bars represent SEM

Anxiety-like behavior was subsequently evaluated using the elevated plus maze. Locomotor activity levels in the EPM were comparable across all groups (all *p* values > 0.5, 2 × 2 two-way ANOVA; Fig. 4A). In the CDPPB experiments, the administration of ketamine had a main effect on the time spent in the open arms (*F*(1,28) = 4.31, *p* = 0.047, *R*^*2*^ = 12.87; Fig. 4B). In the MTEP experiments, a significant interaction was observed between MTEP and ketamine regarding anxiety-like behavior (*F*(1,28) = 15.15, *p* < 0.001, *R*^*2*^ = 34.51, 2 × 2 two-way ANOVA; Fig. 4B). The Ket1 group spent a significantly longer duration in the open arms (*M* = 180.2, *SD* = 131.81) compared to the vehicle group (*M* = 0.53, *SD* = 1.41; *t*(28) = 3.29, *p* = 0.016, *d* = 1.92). Similarly, the MTEP group (*M* = 166.9, *SD* = 141.50) exhibited an increased duration in the open arms compared to the vehicle group (*t*(28) = 3.05, *p* = 0.029, *d* = 1.66). However, the time spent in the open arms of the MTEP + Ket1 group (*M* = 46.3, *SD* = 101.04) did not differ from the vehicle group (*t*(28) = 0.83, *p* = 0.957, Sidak’s corrected; Fig. 4B).

There were no group-level differences in the frequency of unsupported rearing observed in the EPM (all *p* values > 0.5, 2 × 2 two-way ANOVA; Fig. 4C). In contrast, mGluR5 activation via CDPPB resulted in a higher number of supported rearings compared to the vehicle and Ket10 groups (*F*(1,28) = 9.72, *p* < 0.004, *R*^*2*^ = 25.53, 2 × 2 two-way ANOVA; Fig. 4D). In the other set of experiments, MTEP (*F*(1, 28) = 0.53, *p* = 0.471), ketamine (1 mg/kg; *F*(1,28) = 2.05, *p* = 0.163), or their interaction (*F*(1,28) = 2.22, *p* = 0.147) did not yield any difference.

### Fear conditioning and extinction

**Fig. 5:**
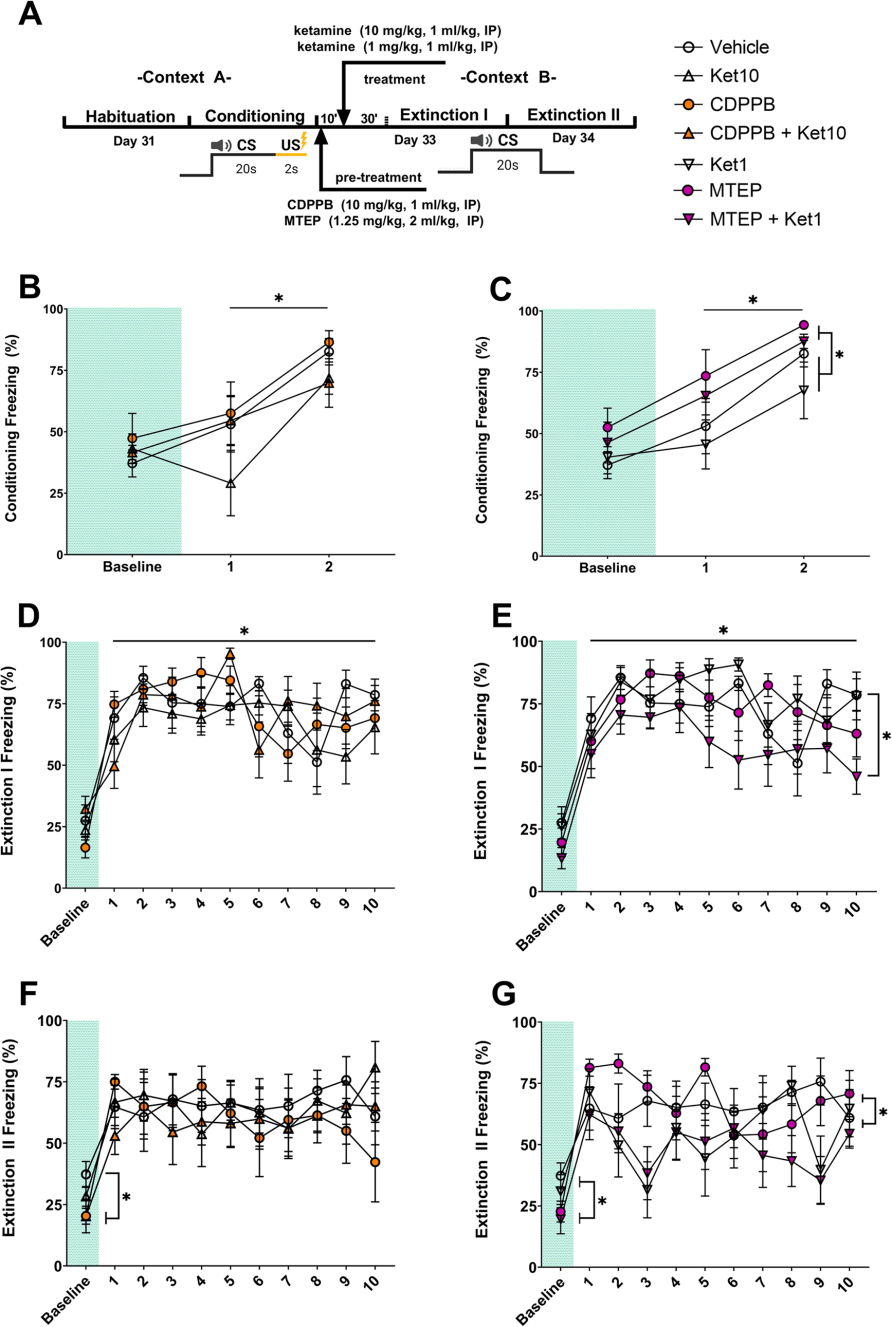
Auditory fear conditioning. **A**) The experimental design and treatment schedule of auditory fear conditioning, and the legend for all experimental groups. **B**) Freezing levels during CS-US pairing in the CDPPB experiments. **C**) Freezing levels during CS-US pairing in the MTEP experiments. **D**) Extinction I freezing levels in the CDPPB experiments. **E**) Extinction I freezing levels in the MTEP experiments. **F**) Extinction II freezing levels in the CDPPB experiments. **G**) Extinction II freezing levels in the MTEP experiments. The same vehicle group was used and plotted for conditioning (**B**, **C**), Extinction I (**D**, **E**), and Extinction II (**F**, **G**). Asterisks denote statistical significance (*p* < .05). Error bars represent SEM

The 3-min acclimation periods of the conditioning (Fig. 5A) were used to record baseline freezing, which were similar for all groups in the CDPPB and MTEP experiments (all *p* values > 0.5, 2 × 2 two-way ANOVA). In the CDPPB experiments, the CS had a main effect on freezing (*F*(1,28) = 19.75, *p* < 0.001, *R*^*2*^ = 21.94, 2 × 2x2 three-way mixed ANOVA; Fig. 5B), indicating that the freezing response towards the cue was increased following the first tone-shock pairing. There was no main effect of the CDPPB (*F*(1,28) = 1.21, *p* = 0.280), ketamine (*F*(1,28) = 3.52, *p* = 0.071) or their interaction (*F*(1,28) = 0.27, *p* = 0.605). Similarly, there was no interaction of the CS with the CDPPB (*F*(1,28) = 1.14, *p* = 0.295), or with ketamine (*F*(1,28) = 0, *p* = 0.985; Fig. 5B).

In the MTEP experiments, a main effect of the CS on freezing was evident (*F*(1,28) = 23.09, *p* < 0.001, *R*^*2*^ = 18.64, 2 × 2x2 three-way mixed ANOVA; Fig. 5C), indicating successful conditioning. Concurrently, a main effect of MTEP was observed (*F*(1,28) = 6.80, *p* = 0.014, *R*^*2*^ = 10.82). However, there was no effect of ketamine (*F*(1,28) = 1.81, *p* = 0.189), and no observable interactions between the CS and ketamine (*F*(1,28) = 0.09, *p* = 0.756), MTEP and ketamine (*F*(1,28) = 0.07, *p* = 0.782; Fig. 5C), or CS and MTEP (*F*(1,28) = 0.193, *p* = 0.664).

In the Extinction session I, baseline freezing levels were similar between the groups in the CDPPB (Fig. 5D) and MTEP experiments (Fig. 5E; all *p* values > 0.5, 2 × 2 two-way ANOVA). In the CDPPB experiments, the main effect for the CS was significant (*F*(9, 252) = 3.17, *p* = 0.001, *R*^*2*^ = 6.71, 2 × 2x10 three-way mixed ANOVA; Fig. 5D), indicating successful fear extinction. There was no interaction of the CS with CDPPB (*F*(9,252) = 1.79, *p* = 0.069), or with ketamine (*F*(9,252) = 1.73, *p* = 0.082). Likewise, we did not observe effects of the CDPPB (*F*(1,28) = 0.30, *p* = 0.584), or ketamine (*F*(1,28) = 0.58, *p* = 0.451) or an interaction (*F*(1,28) = 0.43, *p* = 0.515).

Extinction of the conditioned response was also observed in the MTEP experiments, with a main effect of the CS (*F*(9,252) = 2.74, *p* = 0.004, *R*^*2*^ = 5.83, 2 × 2x10 three-way mixed ANOVA; Fig. 5E). Like the CDPPB experiments, no interaction of the CS and MTEP (*F*(9,252) = 1.72, *p* = 0.084), or CS and ketamine was found (*F*(9,252) = 0.46, *p* = 0.898). No individual main effect of the MTEP (*F*(1,28) = 3.97, *p* = 0.56) or ketamine (*F*(1,28) = 1.45, *p* = 0.237) was observed. However, there was an interaction between the MTEP and ketamine (*F*(1,28) = 4.42, *p* = 0.045, *R*^*2*^ = 3.28, 2 × 2x10 three-way mixed ANOVA; Fig. 5E).

In the Extinction session II, CDPPB administration resulted in lower baseline freezing levels (*F*(1,28) = 6.20, *p* = 0.019, *R*^*2*^ = 17.33, 2 × 2 two-way ANOVA; Fig. 5F). Both the CDPPB (*M* = 20.35, *SD* = 19.33) and the CDPPB + Ket10 (*M* = 20.23, *SD* = 9.14) groups showed lower freezing compared to the vehicle group (*M* = 37.28, *SD* = 14.86; *t*(28) = 2.38, *p* = 0.048, *d* = 0.98 and *t*(28) = 2.40, *p* = 0.046, *d* = 1.38, respectively). Similarly, the MTEP groups also displayed lower baseline freezing levels (*F*(1,28) = 5.32,* p* = 0.029,* R*^*2*^ = 15.63, 2 × 2 two-way ANOVA; Fig. 5G).

Freezing levels in the CDPPB experiments did not change in this session (Fig. 5F; all *p* values > 0.5, 2 × 2x10 three-way mixed ANOVA), indicating lack of further extinction. In the MTEP experiments, ketamine administration exhibited an effect on freezing (*F*(1,28) = 9.06, *p* = 0.005, *R*^*2*^ = 6.14). There was no main effect of the CS (*F*(9,252) = 1.00, *p* = 0.435), or MTEP (*F*(1,28) = 0.06, *p* = 0.793), and no interaction of the MTEP and ketamine (*F*(1,28) = 0.60, *p* = 0.443), CS and MTEP (*F*(9,252) = 1.29, *p* = 0.239), or CS and ketamine (*F*(9,252) = 1.71, *p* = 0.086).

Finally, we compared the average freezing levels of the two extinction sessions. Overall freezing decreased from Extinction I to Extinction II in both the CDPPB (*F*(1,28) = 14.62, *p* < 0.001, *R*^*2*^ = 9.55, 2 × 2x2 three-way mixed ANOVA) and MTEP experiments (*F*(1,28) = 20.15,* p* < 0.001, *R*^*2*^ = 13.38, 2 × 2x2 three-way mixed ANOVA), indicating a significantly better extinction in the second session. There was no group-level difference in the CDPPB experiments (all *p* values > 0.5, 2 × 2x2 three-way mixed ANOVA). However, in the MTEP experiments, the Ket1 and MTEP + Ket1 groups demonstrated a greater reduction in freezing during Extinction II when compared to the MTEP only and vehicle groups (F(1,28) = 6.56, p = 0.016, *R*^*2*^ = 10.68; 2 × 2x2 three-way ANOVA).

## Discussion

This study investigated the impact of mGluR5 on the antidepressant, anxiolytic and fear memory-related effects of ketamine. We activated mGluR5 by using its positive allosteric modulator CDPPB to elucidate its role in the ameliorative effects of an antidepressant dose of ketamine. Subsequently, we evaluated the effects of mGluR5 inhibition by combining low doses of MTEP and ketamine. The results revealed that the antidepressant effect of ketamine was occluded by PAM activation of the mGluR5. In contrast, blocking this receptor with MTEP shortly before administering ketamine produced a synergistic antidepressant effect. However, the combined administration of low doses of MTEP and ketamine nullified the individual anxiolytic effects observed in both the ketamine and MTEP groups 24 h following drug treatment.

The prerequisite of this study was to identify an antidepressant dose of ketamine as well as a sub-effective dose that would not by itself lead to antidepressant-like activity (i.e. significant decrease in overall immobility) in the FST. Utilizing a non/sub-antidepressant dose of ketamine in the second set of experiments was crucial to avoid a ceiling effect and be able to observe any synergistic action of ketamine with MTEP. In the first set of experiments, the 10 mg/kg dose of ketamine did produce an antidepressant effect as observed in several previous studies (Carreno et al. 2016; Choi et al. 2016; Deyama et al. 2019; Engin et al. 2009; Garcia et al. 2008). The 1 mg/kg dose, instead, did not alter behavior in the FST (see Koike and Chaki 2014; Liu et al. 2013; Podkowa et al. 2016), but produced an anxiolytic effect in the EPM.

The CDPPB pre-treatment occluded the antidepressant effects of ketamine, as observed in the deletion or transient inhibition of several molecular players (refer to Table 1 in Kim et al. 2023). It is important to note that CDPPB and MTEP were administered 10 min before IP ketamine in our experiments to ensure an overlap of the peak effects of both drugs. Therefore, the pre-treatment with mGluR5 agents in our study constitutes a concomitant administration protocol, resulting in a partial attenuation or occlusion of ketamine’s antidepressant effect compared to the vehicle-treated animals. However, there was no statistical difference between the Ket10 and CDPPB + Ket10 groups, indicating that CDPPB did not completely prevent the therapeutic action. The occlusion effect may have arisen from the indirect downregulation of the AMPA receptor and NMDAR inhibition via Homer1a, as mentioned earlier (Bockaert et al. 2021). Ketamine upregulates AMPA receptors, and its antidepressant effect can be blocked by pre-treatment with NBQX, an AMPA receptor antagonist (Aleksandrova et al. 2017). Downregulation of the AMPA receptor by positive allosteric modulators of the mGluR5 may have contributed to the occlusion of the antidepressant effect observed in this study. Another factor can be the mGluR5 associated inhibition of the NMDAR activity (Bertaso et al. 2010), as subsequent activation of this receptor was demonstrated to be required for the ketamine’s antidepressant effect (Zanos et al. 2023).

In MTEP experiments, the opposite behavioral effect was observed by combining a low dose of this antagonist with a low, sub-effective dose of ketamine. The behavioral effects of MTEP are typically dose-dependent, similar to those of ketamine. The administration of MTEP at high doses produced antidepressant effects in earlier studies (Domin et al. 2014; Pałucha et al. 2005), while the dose utilized here (1.25 mg/kg) did not affect behavioral despair as confirmed by a previous study (Belozertseva et al. 2007). Combining the sub-effective doses of MTEP and ketamine, however, produced a remarkable synergistic antidepressant effect. The role of mGluR5 in the antidepressant effects of ketamine likely emerge from its interaction with the NMDAR. In an earlier study, pre-treatment with NMDA prevented the antidepressant effects of MTEP, suggesting a functional coupling between these receptors (Pomierny-Chamioło et al. 2010). It must be noted that the MTEP possesses a very limited off-target binding affinity to non-mGluR5 targets including the NMDAR (Lea and Faden 2006), and the aforementioned findings likely emerge due to receptor-receptor interactions between the NMDAR and mGluR5. The FST results of the present study altogether indicate an inverse relationship between mGluR5 activation and the antidepressant effects of ketamine. Suppressed mGluR5 activity is required for the rapid antidepressant action of ketamine, while activation of the mGluR5 occludes this therapeutic effect. More importantly, mGluR5 antagonism can be used to enhance the antidepressant potential of ketamine.

Similar to MTEP, Basimglurant, another mGluR5 antagonist, initially demonstrated promising antidepressant and anxiolytic properties in rodents (Lindemann et al. 2015). However, it did not succeed in the second phase of clinical trials, showing no improvements in mood compared to the placebo group (Youssef et al. 2018). Despite its failure as a stand-alone treatment, Basimglurant showed potential as an adjunctive therapy to monoaminergic antidepressant treatment (SSRIs and SNRIs) in patient-rated measures (Quiroz et al. 2016). These findings suggest that solely blocking mGluR5 may not be sufficient for an effective antidepressant action in humans. However, combining an mGluR5 antagonist with an atypical antidepressant may be beneficial.

The role of mGluR5 in the antidepressant and other therapeutic actions of ketamine will likely depend on its localization within specific cell types. The rapid antidepressant action of ketamine was associated with disinhibition of pyramidal neurons through NMDAR antagonism in GABAergic interneurons (Zanos and Gould 2018), as observed in the medial prefrontal cortex (Zhang et al. 2021) and the hippocampus (Widman and McMahon 2018). Accordingly, mGluR5 antagonism may contribute to antidepressant action of ketamine via modulation of GABAergic interneuron excitability (Chaki et al. 2013). In line with this, the knockout of mGluR5 in glutamatergic neurons induced depressive-like behavior, while the specific knockout of this receptor in GABAergic neurons resulted in an antidepressant effect (Lee et al. 2015). In another study, deletion of the mGluR5 in excitatory neurons of the forebrain did not alter the antidepressant effects of ketamine (Holz et al. 2019). These finding suggest that the CDPPB driven occlusion of the antidepressant effect observed in this study may have emerged via modulation of GABAergic interneurons.

In addition to its synergistic antidepressant action, antagonism of the mGluR5 shortly before ketamine administration abolished the subsequent anxiolytic effects observed in the saline + ketamine and MTEP + saline groups. Importantly, the low dose (1 mg/kg) ketamine utilized in this study produced a significant anxiolytic effect in the EPM. Earlier studies have shown the anxiolytic effects of relatively low doses of ketamine with repeated administration (Horsley et al. 2018; Zhang et al. 2015). Similar anxiolytic outcomes have been observed shortly after the single infusion of 0.1 mg/kg ketamine in healthy subjects (Krystal et al. 1994). A similar level of anxiolytic effect was also observed with the low dose MTEP. In an earlier study, the same dose of MTEP (1.25 mg/kg) administered 30 min prior to testing did not influence anxiety-like behavior in the EPM (Pietraszek et al. 2005b). In contrast, the anxiolytic action of MTEP in this study was observed the day after drug administration, and represents a potentially late onset therapeutic effect on anxiety. In an earlier work, the role of mGluR5 in anxiety was explored in mGluR5-knockout mice, which exhibited anxiety-like phenotypes (Brodkin et al. 2002). In the same study, a high dose of MTEP (16 mg/kg, SC) was found to reduce stress-induced hyperthermia in wild-type animals but had no effect in mGluR5-knockout mice (Brodkin et al. 2002). In contrast, a recent study demonstrated anxiety-like behavior following the knockdown of mGluR5 in CA1 pyramidal neurons (Li et al. 2023), highlighting the differential contribution of mGluR5 localized in various cell types and circuits.

It is important to note that this study is unable to directly compare the behavioral despair results assessed immediately after drug administration with the findings on anxiety obtained 24 h following drug treatment in the EPM. Indeed, the anxiolytic effect of the selective mGluR5 antagonism employed in this study may have been initially latent, as it was not observed in the OFT. Alternatively, the OFT conducted on the same day following drug administration may not have established the necessary conditions to differentiate the experimental groups based on anxiety-like behavior. Animals displayed considerable thigmotaxis in the OFT and spent more time in the periphery of the maze irrespective of their locomotor activity levels. However, in the EPM, a more robust anxiety measure, the vehicle-treated animals exclusively remained in the closed arms, exhibiting a high level of anxiety. In contrast, the experimental groups displayed a roughly bimodal behavioral pattern, with each animal predominantly staying either in the closed or open arms. Notably, this behavior was not attributed to freezing, as all groups displayed sufficient locomotor activity on the maze.

In line with the findings of this study, it has been suggested that the EPM and OFT may be differentially sensitive to anxiolytics and measure distinct aspects of anxiety (Prut and Belzung 2003; Treit et al. 2010). For instance, in a previous study, a high dose of ketamine (50 mg/kg) produced an anxiolytic effect in the EPM but not in the OFT (Engin et al. 2009). Conversely, the anxiogenic effect of GPR30 was observed in the OFT but not in the EPM (Anchan et al. 2014). In this study, neutralizing the individual anxiolytic effects of a low dose of ketamine and MTEP through their combination implies that the NMDAR antagonism by ketamine and the mGluR5 antagonism by MTEP reduce anxiety through distinct molecular pathways, likely involving the activation of the other receptor. Concomitant administration of both agents may have interfered each other’s molecular cascades, blocking or counteracting the resulting anxiolytic effects of each pathway.

There was no effect of the low or high dose ketamine on auditory fear conditioning or extinction, as opposed to earlier observations that report an effect of 10 mg/kg ketamine (IP) on the facilitation of extinction learning (Girgenti et al. 2017; Radford et al. 2018). However, we observed that the concomitant administration of MTEP and ketamine (1 mg/kg) rapidly diminished freezing responses in the first extinction session compared to the vehicle group.

Previous research has shown that higher doses of MTEP (2.5 mg/kg and 5 mg/kg) but not the lower dose (1.25 mg/kg) reduce freezing in an extinction session conducted 24 h after conditioning (Gravius et al. 2006). Our study, using the same low dose of MTEP, illustrated that ketamine enhances the impact of mGluR5 antagonism on fear extinction initiated 24 h after conditioning. Since MTEP also resulted in increased freezing during conditioning, this indicates that a low dose of MTEP plays a general beneficial role in affective memory, as seen in both the acquisition of cued fear memory and extinction learning.

In Extinction II, both the CDPPB and MTEP groups displayed reduced baseline freezing. In earlier observations, the activation of mGluR5 via CDPPB before extinction resulted in decreased freezing in the retention test of contextual conditioning (Sethna and Wang 2014). Likewise, animals administered with MTEP (1.25 mg/kg) showed lower freezing to the context following fear conditioning (Pietraszek et al. 2005b). In our study, the repeated presentation of the CS during Extinction I was carried out in the same context as Extinction II (i.e. Context B). We hypothesized that this might have caused contextual conditioning, leading to a decrease in baseline freezing at the start of Extinction II. Additionally, the experimental groups receiving 1 mg/kg of ketamine exhibited reduced freezing responses throughout Extinction II, possibly due to the delayed increase in locomotor activity induced by this dose of ketamine, as discussed below.

Locomotor activity in an open field was evaluated twice during the experiments. Initially, the animals were placed in the OFT 6 h after the first drug injections on Day 9. This assessment aimed to capture relatively late-onset or sustained alterations in locomotor activity resulting from the pharmacological treatments. On Day 33, during the habituation period of Extinction I, conducted in a novel open arena immediately after the second round of IP injections, we assessed the rapid-onset, acute effects of the drugs on locomotor activity. Notably, none of the groups exhibited changes in locomotor activity during the habituation phase of Extinction I. However, in the OFT, the administration of low dose ketamine resulted in elevated hyperlocomotion, a phenomenon that was normalized by pre-treatment with MTEP. Notably, earlier observations have shown that typically high doses of ketamine (Liu et al. 2013; Razoux et al. 2007), but not the lower doses (Hetzler and Swain Wautlet 1985; Liu et al. 2013; Podkowa et al. 2016), are associated with increased locomotor activity. Nevertheless, a study revealed heightened locomotor activity after repeated administration of a low dose of ketamine (0.5 mg/kg) in the open arms of the EPM 48 h later, which was not observed when a higher dose of ketamine (3 mg/kg) was administered (Horsley et al. 2018). Furthermore, in earlier work, mGluR5 antagonism augmented the hyperlocomotion led by blocking of the NMDAR transmembrane channel via PCP (Henry 2002) or MK-801 (Henry 2002; Pietraszek et al. 2005a).

The anxiolytic dose of ketamine utilized in this study also resulted in a significantly higher frequency of supported rearing in the OFT. However, this effect was not observed in the MTEP + Ket1 group. Unsupported rearing in an open field is linked to acute stress, whereas supported rearing is a common response to a novel environment (Sturman et al. 2018). The increased supported rearing seen in the low dose ketamine group is therefore consistent with the heightened locomotion noted in this group.

The present study revealed the interplay between mGluR5 and the antidepressant and anxiolytic effects of ketamine. Positive allosteric modulation of mGluR5 impeded the antidepressant effect of ketamine, while antagonism of this receptor prior to ketamine administration synergistically enhanced the antidepressant impact. Notably, the combination of mGluR5 antagonism and ketamine nullified the anxiolytic effect independently produced by each of these drugs, indicating a distinctive relationship between NMDAR and mGluR5 in the neural circuits underlying depressive vs. anxiety-like behaviors. In conclusion, our study indicates that inhibiting mGluR5 activity can enhance ketamine’s antidepressant effectiveness. This approach holds promise for fostering the development of innovative cocktail drugs targeting both ionotropic and metabotropic glutamate receptors in depression treatment.

## Data Availability

Data will be made available on request.

## References

[CR1] Akan M, Skorodumov I, Meinhardt MW, Canbeyli R, Unal G (2023). A shea butter-based ketamine ointment: The antidepressant effects of transdermal ketamine in rats. Behav Brain Res.

[CR2] Alagarsamy S, Marino MJ, Rouse ST, Gereau RW, Heinemann SF, Conn PJ (1999). Activation of NMDA receptors reverses desensitization of mGluR5 in native and recombinant systems. Nat Neurosci.

[CR3] Alagarsamy S, Rouse ST, Junge C, Hubert GW, Gutman D, Smith Y, Conn PJ (2002). NMDA-induced phosphorylation and regulation of mGluR5. Pharmacol Biochem Behav.

[CR4] Alagarsamy S, Saugstad J, Warren L, Mansuy IM, Gereau RW, Conn PJ (2005). NMDA-induced potentiation of mGluR5 is mediated by activation of protein phosphatase 2B/calcineurin. Neuropharmacology.

[CR5] Aleksandrova LR, Phillips AG, Wang YT (2017). Antidepressant effects of ketamine and the roles of AMPA glutamate receptors and other mechanisms beyond NMDA receptor antagonism. J Psychiatry Neurosci.

[CR6] Amiri S, Haj-Mirzaian A, Rahimi-Balaei M, Razmi A, Kordjazy N, Shirzadian A, Ejtemaei Mehr S, Sianati H, Dehpour AR (2015). Co-occurrence of anxiety and depressive-like behaviors following adolescent social isolation in male mice; possible role of nitrergic system. Physiol Behav.

[CR7] Anchan D, Clark S, Pollard K, Vasudevan N (2014). GPR30 activation decreases anxiety in the open field test but not in the elevated plus maze test in female mice. Brain and Behavior.

[CR8] Arabzadeh S, Hakkikazazi E, Shahmansouri N, Tafakhori A, Ghajar A, Jafarinia M, Akhondzadeh S (2018). Does oral administration of ketamine accelerate response to treatment in major depressive disorder? Results of a double-blind controlled trial. J Affect Disord.

[CR9] Awad H, Hubert GW, Smith Y, Levey AI, Conn PJ (2000). Activation of metabotropic glutamate receptor 5 has direct excitatory effects and potentiates NMDA receptor currents in neurons of the subthalamic nucleus. J Neurosci.

[CR10] Belozertseva I, Kos T, Popik P, Danysz W, Bespalov A (2007). Antidepressant-like effects of mGluR1 and mGluR5 antagonists in the rat forced swim and the mouse tail suspension tests. Eur Neuropsychopharmacol.

[CR11] Benquet P, Gee CE, Gerber U (2002). Two distinct signaling pathways upregulate NMDA receptor responses via two distinct metabotropic glutamate receptor subtypes. J Neurosci.

[CR12] Bertaso F, Roussignol G, Worley P, Bockaert J, Fagni L, Ango F (2010). Homer1a-dependent crosstalk between NMDA and metabotropic glutamate receptors in mouse neurons. PLoS ONE.

[CR13] Bockaert J, Perroy J, Ango F (2021). The complex formed by group I Metabotropic Glutamate Receptor (mGluR) and Homer1a plays a central role in metaplasticity and homeostatic synaptic scaling. J Neurosci.

[CR14] Brodkin J, Bradbury M, Busse C, Warren N, Bristow LJ, Varney MA (2002). Reduced stress-induced hyperthermia in mGluR5 knockout mice. Eur J Neurosci.

[CR15] Burghardt NS, Sullivan GM, McEwen BS, Gorman JM, LeDoux JE (2004). The selective serotonin reuptake inhibitor citalopram increases fear after acute treatment but reduces fear with chronic treatment: a comparison with tianeptine. Biol Psychiat.

[CR16] Carreno FR, Donegan JJ, Boley AM, Shah A, DeGuzman M, Frazer A, Lodge DJ (2016). Activation of a ventral hippocampus-medial prefrontal cortex pathway is both necessary and sufficient for an antidepressant response to ketamine. Mol Psychiatry.

[CR17] Castro CA, Hogan JB, Benson KA, Shehata CW, Landauer MR (1995). Behavioral effects of vehicles: DMSO, ethanol, Tween-20, Tween-80, and emulphor-620. Pharmacol Biochem Behav.

[CR18] Chaki S, Watanabe M (2023). Antidepressants in the post-ketamine era: Pharmacological approaches targeting the glutamatergic system. Neuropharmacology.

[CR19] Chaki S, Ago Y, Palucha-Paniewiera A, Matrisciano F, Pilc A (2013). mGlu2/3 and mGlu5 receptors: Potential targets for novel antidepressants. Neuropharmacology.

[CR20] Chan M-H, Chiu P-H, Sou J-H, Chen H-H (2008). Attenuation of ketamine-evoked behavioral responses by mGluR5 positive modulators in mice. Psychopharmacology.

[CR21] Chen H-H, Liao P-F, Chan M-H (2011). mGluR5 positive modulators both potentiate activation and restore inhibition in NMDA receptors by PKC dependent pathway. J Biomed Sci.

[CR22] Choi M, Lee SH, Chang HL, Son H (2016). Hippocampal VEGF is necessary for antidepressant-like behaviors but not sufficient for antidepressant-like effects of ketamine in rats. Biochim Biophys Acta (BBA) - Mol Basis Dis.

[CR23] Choi KH, Berman RY, Zhang M, Spencer HF, Radford KD (2020). Effects of ketamine on rodent fear memory. Int J Mol Sci.

[CR24] Clifton NE, Trent S, Thomas KL, Hall J (2019). Regulation and function of activity-dependent homer in synaptic plasticity. Complex Psychiatry.

[CR25] Cooper MD, Rosenblat JD, Cha DS, Lee Y, Kakar R, McIntyre RS (2017). Strategies to mitigate dissociative and psychotomimetic effects of ketamine in the treatment of major depressive episodes: a narrative review. World J Biol Psychiatry.

[CR26] Cryan JF, Valentino RJ, Lucki I (2005). Assessing substrates underlying the behavioral effects of antidepressants using the modified rat forced swimming test. Neurosci Biobehav Rev.

[CR27] de Bartolomeis A, Sarappa C, Buonaguro EF, Marmo F, Eramo A, Tomasetti C, Iasevoli F (2013). Different effects of the NMDA receptor antagonists ketamine, MK-801, and memantine on postsynaptic density transcripts and their topography: Role of Homer signaling, and implications for novel antipsychotic and pro-cognitive targets in psychosis. Prog Neuropsychopharmacol Biol Psychiatry.

[CR28] DeLorenzo C, DellaGioia N, Bloch M, Sanacora G, Nabulsi N, Abdallah C, Yang J, Wen R, Mann JJ, Krystal JH, Parsey RV, Carson RE, Esterlis I (2015). In Vivo Ketamine-Induced Changes in [11 C]ABP688 Binding to Metabotropic Glutamate Receptor Subtype 5. Biol Psychiat.

[CR29] Deyama S, Bang E, Wohleb ES, Li X-Y, Kato T, Gerhard DM, Dutheil S, Dwyer JM, Taylor SR, Picciotto MR, Duman RS (2019). Role of Neuronal VEGF Signaling in the Prefrontal Cortex in the Rapid Antidepressant Effects of Ketamine. Am J Psychiatry.

[CR30] Domin H, Szewczyk B, Woźniak M, Wawrzak-Wleciał A, Śmiałowska M (2014). Antidepressant-like effect of the mGluR5 antagonist MTEP in an astroglial degeneration model of depression. Behav Brain Res.

[CR31] Ecevitoglu A, Canbeyli R, Unal G (2019). Oral ketamine alleviates behavioral despair without cognitive impairment in Wistar rats. Behav Brain Res.

[CR32] Engin E, Treit D, Dickson CT (2009). Anxiolytic- and antidepressant-like properties of ketamine in behavioral and neurophysiological animal models. Neuroscience.

[CR33] Esterlis I, DellaGioia N, Pietrzak RH, Matuskey D, Nabulsi N, Abdallah CG, Yang J, Pittenger C, Sanacora G, Krystal JH, Parsey RV, Carson RE, DeLorenzo C (2018). Ketamine-induced reduction in mGluR5 availability is associated with an antidepressant response: an [11C]ABP688 and PET imaging study in depression. Mol Psychiatry.

[CR34] Fraser LM, Brown RE, Hussin A, Fontana M, Whittaker A, O’Leary TP, Lederle L, Holmes A, Ramos A (2010). Measuring anxiety- and locomotion-related behaviours in mice: a new way of using old tests. Psychopharmacology.

[CR35] Fukumoto K, Iijima M, Chaki S (2016). The Antidepressant effects of an mGlu2/3 receptor antagonist and ketamine require AMPA receptor stimulation in the mPFC and subsequent activation of the 5-HT neurons in the DRN. Neuropsychopharmacology.

[CR36] Garcia LSB, Comim CM, Valvassori SS, Réus GZ, Barbosa LM, Andreazza AC, Stertz L, Fries GR, Gavioli EC, Kapczinski F, Quevedo J (2008). Acute administration of ketamine induces antidepressant-like effects in the forced swimming test and increases BDNF levels in the rat hippocampus. Prog Neuropsychopharmacol Biol Psychiatry.

[CR37] Gerhard DM, Pothula S, Liu RJ, Wu M, Li XY, Girgenti MJ, Taylor SR, Duman CH, Delpire E, Picciotto M, Wohleb ES, Duman RS (2020). GABA interneurons are the cellular trigger for ketamine’s rapid antidepressant actions. J Clin Investig.

[CR38] Girgenti MJ, Ghosal S, LoPresto D, Taylor JR, Duman RS (2017). Ketamine accelerates fear extinction via mTORC1 signaling. Neurobiol Dis.

[CR39] Gravius A, Barberi C, Schäfer D, Schmidt WJ, Danysz W (2006). The role of group I metabotropic glutamate receptors in acquisition and expression of contextual and auditory fear conditioning in rats – a comparison. Neuropharmacology.

[CR40] Henry S (2002). The mGluR5 antagonist MPEP, but not the mGluR2/3 agonist LY314582, augments PCP effects on prepulse inhibition and locomotor activity. Neuropharmacology.

[CR41] Hetzler BE, Swain Wautlet B (1985). Ketamine-induced locomotion in rats in an open-field. Pharmacol Biochem Behav.

[CR42] Holz A, Mülsch F, Schwarz MK, Hollmann M, Döbrössy MD, Coenen VA, Bartos M, Normann C, Biber K, van Calker D, Serchov T (2019). Enhanced mGlu5 signaling in excitatory neurons promotes rapid antidepressant effects via AMPA receptor activation. Neuron.

[CR43] Horsley RR, Páleníček T, Kolin J, Valeš K (2018). Psilocin and ketamine microdosing: effects of subchronic intermittent microdoses in the elevated plus-maze in male Wistar rats. Behav Pharmacol.

[CR44] Jesse CR, Bortolatto CF, Savegnago L, Rocha JB, Nogueira CW (2008). Involvement of L-arginine-nitric oxide-cyclic guanosine monophosphate pathway in the antidepressant-like effect of tramadol in the rat forced swimming test. Prog Neuropsychopharmacol Biol Psychiatry.

[CR45] Jin D, Guo M, Xue B, Mao L, Wang JQ (2013). Differential regulation of CaMKIIα interactions with mGluR5 and NMDA receptors by Ca(2+) in neurons. J Neurochem.

[CR46] Kim J-W, Suzuki K, Kavalali ET, Monteggia LM (2023). Bridging rapid and sustained antidepressant effects of ketamine. Trends Mol Med.

[CR47] Kingir E, Sevinc C, Unal G (2023). Chronic oral ketamine prevents anhedonia and alters neuronal activation in the lateral habenula and nucleus accumbens in rats under chronic unpredictable mild stress. Neuropharmacology.

[CR48] Koike H, Chaki S (2014). Requirement of AMPA receptor stimulation for the sustained antidepressant activity of ketamine and LY341495 during the forced swim test in rats. Behav Brain Res.

[CR49] Koike H, Iijima M, Chaki S (2011). Involvement of AMPA receptor in both the rapid and sustained antidepressant-like effects of ketamine in animal models of depression. Behav Brain Res.

[CR50] Kokras N, Polissidis A, Antoniou K, Dalla C (2017). Head shaking in the forced swim test: A robust but unexplored sex difference. Pharmacol Biochem Behav.

[CR51] Konieczny J, Ossowska K, Wolfarth S, Pilc A (1998). LY354740, a group II metabotropic glutamate receptor agonist with potential antiparkinsonian properties in rats. Naunyn Schmiedebergs Arch Pharmacol.

[CR52] Krystal JH, Mathew SJ, D’Souza DC, Garakani A, Gunduz-Bruce H, Charney DS (2010). Potential psychiatric applications of metabotropic glutamate receptor agonists and antagonists. CNS Drugs.

[CR53] Krystal JH, Karper LP, Seibyl JP, Freeman GK, Delaney R, Bremner JD, Heninger GR, Bowers MB Jr, Charney DS (1994) Subanesthetic effects of the noncompetitive NMDA antagonist, ketamine, in humans. Psychotomimetic, perceptual, cognitive, and neuroendocrine responses. Arch Gen Psychiatry 51(3):199–214. 10.1001/archpsyc.1994.03950030035004 10.1001/archpsyc.1994.03950030035004 8122957

[CR54] Lapidus KAB, Levitch CF, Perez AM, Brallier JW, Parides MK, Soleimani L, Feder A, Iosifescu DV, Charney DS, Murrough JW (2014). A randomized controlled trial of intranasal ketamine in major depressive disorder. Biol Psychiat.

[CR55] Lea PM, Faden AI (2006). Metabotropic glutamate receptor subtype 5 antagonists MPEP and MTEP. CNS Drug Rev.

[CR56] Lee K-W, Westin L, Kim J, Chang JC, Oh Y-S, Amreen B, Gresack J, Flajolet M, Kim D, Aperia A, Kim Y, Greengard P (2015). Alteration by p11 of mGluR5 localization regulates depression-like behaviors. Mol Psychiatry.

[CR57] Li Y, Zhu ZR, Ou BC, Wang YQ, Tan ZB, Deng CM, Gao YY, Tang M, So JH, Mu YL, Zhang LQ (2015). Dopamine D2/D3 but not dopamine D1 receptors are involved in the rapid antidepressant-like effects of ketamine in the forced swim test. Behav Brain Res.

[CR58] Li X, Du ZJ, Xu JN, Liang ZM, Lin S, Chen H, Li SJ, Li XW, Yang JM, Gao TM (2023). mGluR5 in hippocampal CA1 pyramidal neurons mediates stress-induced anxiety-like behavior. Neuropsychopharmacology.

[CR59] Lindemann L, Porter RH, Scharf SH, Kuennecke B, Bruns A, von Kienlin M, Harrison AC, Paehler A, Funk C, Gloge A, Schneider M, Parrott NJ, Polonchuk L, Niederhauser U, Morairty SR, Kilduff TS, Vieira E, Kolczewski S, Wichmann J, Hartung T, … Jaeschke G (2015) Pharmacology of Basimglurant (RO4917523, RG7090), a unique metabotropic glutamate receptor 5 negative allosteric modulator in clinical development for depression. J Pharmacol Exp Ther 353(1):213–233. 10.1124/jpet.114.222463 10.1124/jpet.114.222463 25665805

[CR60] Liu R-J, Fuchikami M, Dwyer JM, Lepack AE, Duman RS, Aghajanian GK (2013). GSK-3 inhibition potentiates the synaptogenic and antidepressant-like effects of subthreshold doses of ketamine. Neuropsychopharmacology.

[CR61] Maeng S, Zarate CA, Du J, Schloesser RJ, McCammon J, Chen G, Manji HK (2008). Cellular mechanisms underlying the antidepressant effects of ketamine: Role of α-Amino-3-Hydroxy-5-Methylisoxazole-4-Propionic acid receptors. Biol Psychiat.

[CR62] Matta JA, Ashby MC, Sanz-Clemente A, Roche KW, Isaac JTR (2011). mGluR5 and NMDA receptors drive the experience- and activity-dependent NMDA receptor NR2B to NR2A subunit switch. Neuron.

[CR63] Niswender CM, Conn PJ (2010). Metabotropic glutamate receptors: Physiology, pharmacology, and disease. Annu Rev Pharmacol Toxicol.

[CR64] Pałucha A, Brański P, Szewczyk B, Wierońska JM, Kłak K, Pilc A (2005). Potential antidepressant-like effect of MTEP, a potent and highly selective mGluR5 antagonist. Pharmacol Biochem Behav.

[CR65] Pałucha-Poniewiera A, Podkowa K, Pilc A (2019). Role of AMPA receptor stimulation and TrkB signaling in the antidepressant-like effect of ketamine co-administered with a group II mGlu receptor antagonist, LY341495, in the forced swim test in rats. Behav Pharmacol.

[CR66] Pennington ZT, Dong Z, Feng Y, Vetere LM, Page-Harley L, Shuman T, Cai DJ (2019). ezTrack: An open-source video analysis pipeline for the investigation of animal behavior. Sci Rep.

[CR67] Pietraszek M, Gravius A, Schäfer D, Weil T, Trifanova D, Danysz W (2005). mGluR5, but not mGluR1, antagonist modifies MK-801-induced locomotor activity and deficit of prepulse inhibition. Neuropharmacology.

[CR68] Pietraszek M, Sukhanov I, Maciejak P, Szyndler J, Gravius A, Wisłowska A, Płaźnik A, Bespalov AY, Danysz W (2005). Anxiolytic-like effects of mGlu1 and mGlu5 receptor antagonists in rats. Eur J Pharmacol.

[CR69] Piva A, Caffino L, Padovani L, Pintori N, Mottarlini F, Sferrazza G, Paolone G, Fumagalli F, Chiamulera C (2020). The metaplastic effects of ketamine on sucrose renewal and contextual memory reconsolidation in rats. Behav Brain Res.

[CR70] Podkowa K, Pochwat B, Brański P, Pilc A, Pałucha-Poniewiera A (2016). Group II mGlu receptor antagonist LY341495 enhances the antidepressant-like effects of ketamine in the forced swim test in rats. Psychopharmacology.

[CR71] Pomierny-Chamioło L, Poleszak E, Pilc A, Nowak G (2010). NMDA but not AMPA glutamatergic receptors are involved in the antidepressant-like activity of MTEP during the forced swim test in mice. Pharmacol Rep.

[CR72] Porsolt RD, Le Pichon M, Jalfre M (1977). Depression: a new animal model sensitive to antidepressant treatments. Nature.

[CR73] Prut L, Belzung C (2003). The open field as a paradigm to measure the effects of drugs on anxiety-like behaviors: a review. Eur J Pharmacol.

[CR74] Quiroz JA, Tamburri P, Deptula D, Banken L, Beyer U, Rabbia M, Parkar N, Fontoura P, Santarelli L (2016). Efficacy and safety of Basimglurant as adjunctive therapy for major depression: A randomized clinical trial. JAMA Psychiat.

[CR75] Radford KD, Park TY, Jaiswal S, Pan H, Knutsen A, Zhang M, Driscoll M, Osborne-Smith LA, Dardzinski BJ, Choi KH (2018). Enhanced fear memories and brain glucose metabolism (18F-FDG-PET) following sub-anesthetic intravenous ketamine infusion in Sprague-Dawley rats. Transl Psychiatry.

[CR76] Razoux F, Garcia R, Léna I (2007). Ketamine, at a Dose that disrupts motor behavior and latent inhibition, enhances prefrontal cortex synaptic efficacy and glutamate release in the nucleus accumbens. Neuropsychopharmacology.

[CR77] Romano C, Sesma MA, McDonald CT, O’malley K, van den Pol AN, Olney JW (1995). Distribution of metabotropic glutamate receptor mGluR5 immunoreactivity in rat brain. J Comp Neurol.

[CR78] Serchov T, Clement H-W, Schwarz MK, Iasevoli F, Tosh DK, Idzko M, Jacobson KA, de Bartolomeis A, Normann C, Biber K, van Calker D (2015). Increased signaling via adenosine A1 receptors, sleep deprivation, imipramine, and ketamine inhibit depressive-like behavior via induction of homer1a. Neuron.

[CR79] Sethna F, Wang H (2014). Pharmacological enhancement of mGluR5 facilitates contextual fear memory extinction. Learn Mem.

[CR80] Silote GP, de Oliveira SFS, Ribeiro DE, Machado MS, Andreatini R, Joca SRL, Beijamini V (2020). Ketamine effects on anxiety and fear-related behaviors: Current literature evidence and new findings. Prog Neuropsychopharmacol Biol Psychiatry.

[CR81] Sofia RD, Harakal JD (1975). Evaluation of ketamine HCl for anti-depressant activity. Arch Int Pharmacodyn Ther.

[CR82] Sou J-H, Chan M-H, Chen H-H (2006). Ketamine, but not propofol, anaesthesia is regulated by metabotropic glutamate 5 receptors. Br J Anaesth.

[CR83] Sturman O, Germain P-L, Bohacek J (2018). Exploratory rearing: a context- and stress-sensitive behavior recorded in the open-field test. Stress.

[CR84] Su LD, Wang N, Han J, Shen Y (2022). Group 1 Metabotropic glutamate receptors in neurological and psychiatric diseases: Mechanisms and prospective. Neuroscientist.

[CR85] Tanyeri P, Buyukokuroglu ME, Mutlu O, Ulak G, Yıldız Akar F, Komsuoglu Celikyurt I, Erden BF (2013). Involvement of serotonin receptor subtypes in the antidepressant-like effect of beta receptor agonist Amibegron (SR 58611A): an experimental study. Pharmacol Biochem Behav.

[CR86] Treit D, Engin E, McEown K (2010). Animal models of anxiety and anxiolytic drug action. Curr Top Behav Neurosci.

[CR87] Truppman Lattie D, Nehoff H, Neehoff S, Gray A, Glue P (2021). Anxiolytic effects of acute and maintenance ketamine, as assessed by the fear questionnaire subscales and the Spielberger State anxiety rating scale. J Psychopharmacol.

[CR88] Tu JC, Xiao B, Naisbitt S, Yuan JP, Petralia RS, Brakeman P, Doan A, Aakalu VK, Lanahan AA, Sheng M, Worley PF (1999). Coupling of mGluR/Homer and PSD-95 complexes by the shank family of postsynaptic density proteins. Neuron.

[CR89] Unal G, Canbeyli R (2019). Psychomotor retardation in depression: A critical measure of the forced swim test. Behav Brain Res.

[CR90] Valle FP (1970). Effects of strain, sex, and illumination on open-field behavior of rats. Am J Psychol.

[CR91] Walf AA, Frye CA (2007). The use of the elevated plus maze as an assay of anxiety-related behavior in rodents. Nat Protoc.

[CR92] Wang Y, He W, Zhang H, Yao Z, Che F, Cao Y, Sun H (2020). mGluR5 mediates ketamine antidepressant response in susceptible rats exposed to prenatal stress. J Affect Disord.

[CR93] Widman AJ, McMahon LL (2018) Disinhibition of CA1 pyramidal cells by low-dose ketamine and other antagonists with rapid antidepressant efficacy. Proc Natl Acad Sci 115(13). 10.1073/pnas.1718883115 10.1073/pnas.1718883115 PMC5879689 29531088

[CR94] Yankelevitch-Yahav R, Franko M, Huly A, Doron R (2015). The forced swim test as a model of depressive-like behavior. J Vis Exp.

[CR95] Yilmaz A, Schulz D, Aksoy A, Canbeyli R (2002). Prolonged effect of an anesthetic dose of ketamine on behavioral despair. Pharmacol Biochem Behav.

[CR96] Youssef EA, Berry-Kravis E, Czech C, Hagerman RJ, Hessl D, Wong CY, Rabbia M, Deptula D, John A, Kinch R, Drewitt P, Lindemann L, Marcinowski M, Langland R, Horn C, Fontoura P, Santarelli L, Quiroz JA, FragXis Study Group (2018). Effect of the mGluR5-NAM Basimglurant on behavior in adolescents and adults with fragile X syndrome in a randomized, double-blind, placebo-controlled trial: FragXis phase 2 results. Neuropsychopharmacology.

[CR97] Zanos P, Gould TD (2018). Mechanisms of ketamine action as an antidepressant. Mol Psychiatry.

[CR98] Zanos P, Highland JN, Stewart BW, Georgiou P, Jenne CE, Lovett J, Morris PJ, Thomas CJ, Moaddel R, Zarate CA, Gould TD (2019). 2R,6R hydroxynorketamine exerts mGlu2/3 receptor-dependent antidepressant actions. Proc Natl Acad Sci.

[CR99] Zanos P, Brown KA, Georgiou P, Yuan P, Zarate CA, Thompson SM, Gould TD (2023). NMDA receptor activation-dependent antidepressant-relevant behavioral and synaptic actions of ketamine. J Neurosci.

[CR100] Zarate CA, Singh JB, Carlson PJ, Brutsche NE, Ameli R, Luckenbaugh DA, Charney DS, Manji HK (2006). A randomized trial of an N-methyl-D-aspartate antagonist in treatment-resistant major depression. Arch Gen Psychiatry.

[CR101] Zhang LM, Zhou WW, Ji YJ, Li Y, Zhao N, Chen HX, Xue R, Mei XG, Zhang YZ, Wang HL, Li YF (2015). Anxiolytic effects of ketamine in animal models of posttraumatic stress disorder. Psychopharmacology.

[CR102] Zhang K, Yamaki VN, Wei Z, Zheng Y, Cai X (2017). Differential regulation of GluA1 expression by ketamine and memantine. Behav Brain Res.

[CR103] Zhang B, Yang X, Ye L, Liu R, Ye B, Du W, Shen F, Li Q, Guo F, Liu J, Guo F, Li Y, Xu Z, Liu Z (2021). Ketamine activated glutamatergic neurotransmission by GABAergic disinhibition in the medial prefrontal cortex. Neuropharmacology.

[CR104] Zhang K, Xu T, Yuan Z, Wei Z, Yamaki VN, Huang M, Huganir RL, Cai X (2016) Essential roles of AMPA receptor GluA1 phosphorylation and presynaptic HCN channels in fast-acting antidepressant responses of ketamine. Sci Signal 9(458). 10.1126/scisignal.aai7884 10.1126/scisignal.aai7884 PMC5564288 27965425

[CR105] Zomkowski AD, Santos AR, Rodrigues AL (2005). Evidence for the involvement of the opioid system in the agmatine antidepressant-like effect in the forced swimming test. Neurosci Lett.

